# The Synthesis Methodology of PEGylated Fe_3_O_4_@Ag Nanoparticles Supported by Their Physicochemical Evaluation

**DOI:** 10.3390/molecules26061744

**Published:** 2021-03-20

**Authors:** Magdalena Kędzierska, Piotr Potemski, Anna Drabczyk, Sonia Kudłacik-Kramarczyk, Magdalena Głąb, Beata Grabowska, Dariusz Mierzwiński, Bożena Tyliszczak

**Affiliations:** 1 Department of Chemotherapy, Medical University of Lodz, WWCOiT Copernicus Hospital, 90-001 Lodz, Poland; kameleonmagda6@gmail.com (M.K.); piotr.potemski@umed.lodz.pl (P.P.); 2 Institute of Materials Science, Faculty of Materials Engineering and Physics, Cracow University of Technology, 37 Jana Pawła II Av., 31-864 Krakow, Poland; magdalena.glab@doktorant.pk.edu.pl (M.G.); dariusz.mierzwinski@pk.edu.pl (D.M.); 3 Faculty of Foundry Engineering, AGH University of Technology, 23 Reymonta St., 30-059 Krakow, Poland; beata.grabowska@agh.edu.pl

**Keywords:** iron (II, III) oxide nanoparticles, Fe_3_O_4_ nanoparticles, silver nanoparticles, poly(ethylene glycol), PEG, Massart synthesis, arabic gum, steric stabilization, sound-energy using sonication, agglomerate disintegration

## Abstract

Many investigations are currently being performed to develop the effective synthesis methodology of magnetic nanoparticles with appropriately functionalized surfaces. Here, the novelty of the presented work involves the preparation of nano-sized PEGylated Fe_3_O_4_@Ag particles, i.e., the main purpose was the synthesis of magnetic nanoparticles with a functionalized surface. Firstly, Fe_3_O_4_ particles were prepared via the Massart process. Next, Ag+ reduction was conducted in the presence of Fe_3_O_4_ particles to form a nanosilver coating. The reaction was performed with arabic gum as a stabilizing agent. Sound energy-using sonication was applied to disintegrate the particles’ agglomerates. Next, the PEGylation process aimed at the formation of a coating on the particles’ surface using PEG (poly(ethylene glycol)) has been performed. It was proved that the arabic gum limited the agglomeration of nanoparticles, which was probably caused by the steric effect caused by the branched compounds from the stabilizer that adsorbed on the surface of nanoparticles. This effect was also enhanced by the electrostatic repulsions. The process of sonication caused the disintegration of aggregates. Formation of iron (II, III) oxide with a cubic structure was proved by diffraction peaks. Formation of a nanosilver coating on the Fe_3_O_4_ nanoparticles was confirmed by diffraction peaks with 2θ values 38.15° and 44.35°. PEG coating on the particles’ surface was proven via FT-IR (Fourier Transform Infrared Spectroscopy) analysis. Obtained PEG–nanosilver-coated Fe_3_O_4_ nanoparticles may find applications as carriers for targeted drug delivery using an external magnetic field.

## 1. Introduction

Nanotechnology is a field dealing with the synthesis of various structures having at least a size within the range 1–100 nm and is one of the most rapidly developing sciences [1]. Nanomaterials find applications in such areas as food packaging [2], the electric industry [3,4], or catalysts [5,6]. Nonetheless, particular interest in nano-sized materials is currently observed in medicine and the related areas, i.e., in orthopedy [7], dentistry [8,9], or in drug delivery [10,11,12].

In the case of drug delivery, particular attention is directed toward gold [13] and magnetic nanoparticles [14]. Magnetic nanoparticles are particularly desirable for such an application due to the possibility of their delivery to the specific place in the organism by the use of an external magnetic field [15]. In order to enable the adequate interaction between such a drug carrier and the selected active substance, a coating based on natural or synthetic compounds is formed on the surface of magnetic nanoparticles [16,17,18]. For example, Ayubi et al. performed investigations on magnetic nanoparticles coated with PEGylated curcumin. Such systems were characterized as biocompatible controlled release carriers at pH 5. [19]. Targeted delivery of drugs by functionalized magnetic nanoparticles is particularly worth investigating in anticancer treatment [20,21]. Xie et al. conducted studies on the fabrication of magnetic nanoparticles as carriers of acyclovir—the active substance known for its anticancer activity [22]. Next, Saepudin et al. reported on various substances used for the modification of magnetic nanoparticles to achieve the effective release of doxorubicin by such formed drug carriers [23]. In the research of Patil et al., magnetic nanoparticles were proposed as carriers of camptothecin—a poorly water-soluble anticancer drug [24]. The improvement of cancer treatment by magnetic nanoparticles may be achieved by combination of the hyperthermia and targeted drug delivery. Such an approach has been proposed, e.g., by Singh et al. They presented the application of these nanoparticles in a dual role, i.e., as a carrier of paclitaxel and as an inducer applied for hyperthermia under the alternating magnetic field. It was proved that such a combined therapy exhibited higher efficiency than that using only one from the mentioned activities [25]. The application potential of magnetic nanoparticles in hyperthermia has been also discussed by Darwish et al. [26], Hammad et al. [27], and Kalubowilage et al. [28].

Apart from wide application of magnetic nanoparticles in drug delivery systems, these nanomaterials find also use in regenerative medicine, tissue engineering or in bone surgery [29]. For example, Tanasa et al. performed investigations on the scaffolds based on silk fibroin and decorated with magnetic nanoparticles. They reported that such scaffolds were biocompatible and affected positively the cell proliferation [30]. Next, Steckiewicz and Inkielewicz–Stepniak discussed the significance of nanomaterials in bone-related issues in their work, including, e.g., the application of selected nanoparticles for the improvement of the biocompatibility of implant surfaces [31].

Due to the growing interest in functionalized magnetic nanoparticles, many investigations are currently being carried out to develop new methods of syntheses of such coated nanomaterials. The main objective of the research was to create PEGylated Fe_3_O_4_@Ag particles having a size between 1 and 100 nm. Such systems have been developed for applications as drug carriers which, due to the presence of nanosilver coatings, are additionally characterized by antimicrobial properties. Importantly, PEGylation of nanoparticles is important due to the fact that this polymer constitutes a linker between a drug and a magnetic core, and additionally prevents the adhesion of proteins or antibodies to such coated nanoparticles [32,33,34]. Additionally, PEGylated particles show slightly higher sizes than those without this layer, which is also beneficial because obtained systems are designed to deliver cytostatics directly to cancer cells. The gaps in the structure of the capillaries of neoplastic tissues have a slightly bigger diameter than the gaps in properly developing issues so numerous particles may diffuse through properly functioning tissues, although only the larger ones—such as PEGylated nanoparticles—may diffuse through the capillaries of cancer tissues [35]. Performed synthesis involved two steps, i.e., preparation of iron oxide nanoparticles via Massart synthesis and their further functionalization by nanosilver and poly(ethylene glycol) PEG. Investigations over the obtained materials included determining their size using the DLS (Dynamic Light Scattering) method, characterization of their chemical structure via FT-IR spectroscopy, and determining their crystallinity by the XRD (X-Ray Diffraction) technique. Additionally, surface morphology analysis supplemented with elemental analysis has been performed. The presence of the nanosilver shell has been verified by UV–Vis (Ultraviolet Visible) spectroscopy and TEM (Transmission Electron Microscopy) analysis.

## 2. Results and Discussion

**Figure 1:**
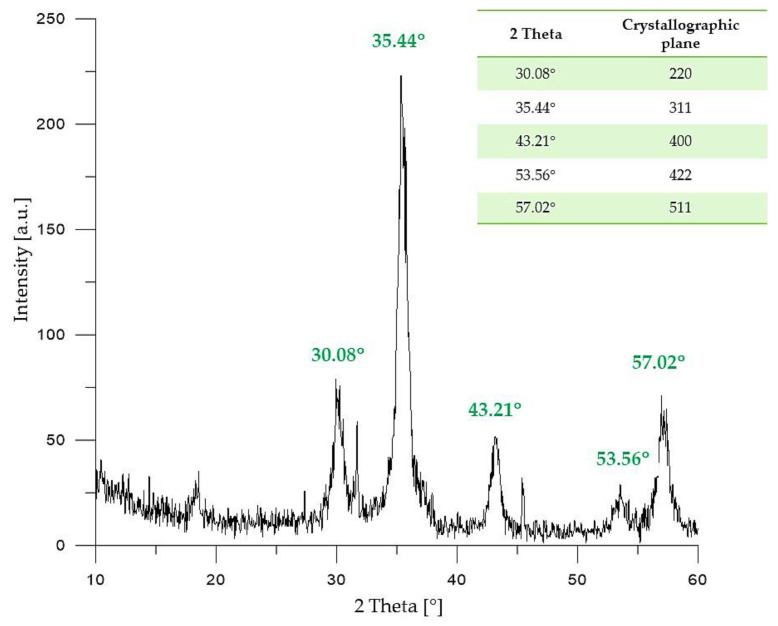
XRD (X-Ray Diffraction) pattern of Fe_3_O_4_ particles.

Crystallinity of Fe_3_O_4_ particles obtained via Massart synthesis was verified using the XRD technique. In Figure 1, the XRD pattern of the material analyzed is presented.

On the X-ray diffractogram of Fe_3_O_4_ particles presented above, five diffraction peaks with 2θ values of 30.08°; 35.44°; 43.21°; 53.56° and 57.02° may be observed. The mentioned peaks are characteristic for the following crystallographic planes of iron (II, III) oxide with a cubic structure: 220, 311, 400, 422, and 511, respectively. The diffraction peaks indicated on the XRD diffractogram presented in Figure 1 correspond to the diffraction peaks of pure Fe_3_O_4_ presented in the reference database (JCPDS Card: 19–629) [36,37], and are very similar to the XRD patterns of Fe_3_O_4_ nanoparticles presented in other publications [38].

**Figure 2:**
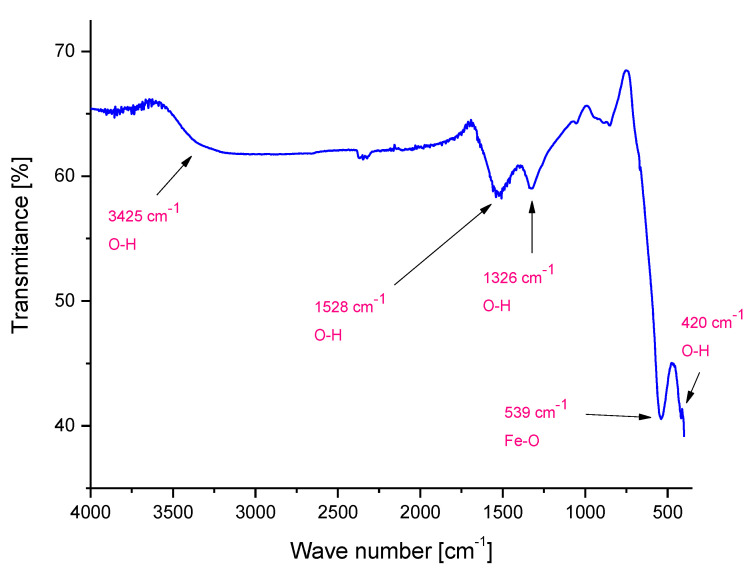
FT-IR (Fourier Transform Infrared Spectroscopy) spectrum of Fe_3_O_4_ particles.

In Figure 2 below, a FT-IR spectrum of the analyzed Fe_3_O_4_ particles is shown.

FT-IR analysis enabled us to determine the presence of characteristic groups in the tested material based on their vibration under the influence of the specific wavelength from the infrared range. On the FT-IR spectrum presented Figure 2, bands at 539 cm^−1^ and 420 cm^−1^ corresponding to the stretching vibrations of Fe–O can be observed [39]. The presence of such bands confirmed the formation of Fe_3_O_4_ particles. Moreover, bands at 1326 cm^−1^, 1528 cm^−1^ and 3425 cm^−1^ are also visible. They derive probably from the hydroxylic groups or from molecules of water—the synthesis of Fe_3_O_4_ particles is performed in an aqueous environment. Hydroxylic groups may be bonded by hydrogen bonds to the surface of iron oxide particles. On the other hand, the molecules of water may be chemically adsorbed on the surface of magnetic particles obtained.

**Figure 3:**
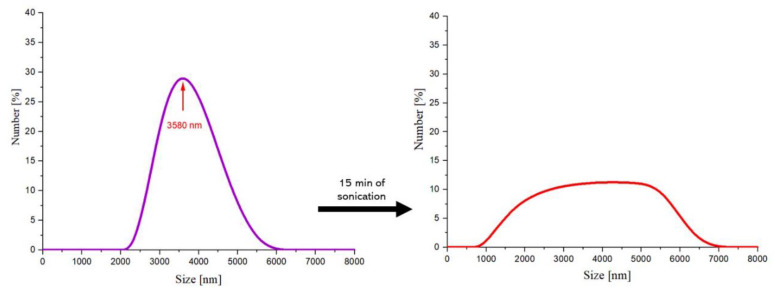
DLS (Dynamic Light Scattering) measurements of the Fe_3_O_4_ particles directly after the synthesis (left) and after 15 min of sonication (right).

In Figure 3, results of the particles size analysis performed via the DLS technique are presented. The size of particles was determined directly after the Massart synthesis and after 15 min of sonication had been applied.

Based on the DLS analysis performed, it can be reported that in the suspension analyzed directly after the Massart synthesis, particles having a size within the range 2000–6000 nm may be present. The largest number of particles—i.e., 30%—had a size of 3580 nm. In general, particles as defined as nanoparticles when at least one from their dimensions is within the range of 1–100 nm. Therefore, it may be reported that the analyzed suspension does not contain nanoparticles, only microparticles. This is probably due to the fact that iron (II, III) oxide nanoparticles (also known as magnetite or magnetic nanoparticles) combine and form agglomerates (aggregates of particles) as a result of their superparamagnetic properties. The low stability of such particles contributes to their agglomeration and this, in turn, is problematic and requires the synthesis performed under appropriate conditions that will limit this process as much as possible.

Suspension of the particles received via Massart synthesis was subjected to the sonication process for 15 min, which was also followed by particles size analysis using the DLS technique. Based on such an investigation, it was stated that particles of approximately 800–7000 nm in size were found in the tested suspension; therefore, it may also be concluded that the nanometric-size particles have still not been obtained. Additionally, a larger size distribution was observed that is probably due to the applied sonication process.

On the one hand, the agglomerates with a size above 6000 nm, i.e., those whose presence have not been reported in the suspension analyzed directly after the Massart synthesis, may be observed in the suspension after the sonication process. This indicates that the particles still agglomerate. On the other hand, a decrease in the number of particles having a size of approximately 4000 nm was also observed (before sonication—30% of total particles, after this process–approximately 10%). Probably, some of the agglomerates have been disintegrated. Moreover, the appearance of particles with a size below 2000 nm has been noticed.

Due to the fact that magnetic nanoparticles tend strongly to agglomerate, which has been demonstrated through the performed investigations, it was decided to carry out further syntheses in the presence of a stabilizing agent, i.e., arabic gum. Such a stabilizer has been chosen because of a wide variety of chemical compounds in its composition which may affect the course of the magnetic nanoparticle synthesis.

Zeta potential (ζ) defines the electrokinetic potential of colloidal systems and constitutes a measure of the surface charge. The measurement of the zeta potential is often used to determine the stability of a suspension. It was proved that the particles with almost neutral zeta potential (i.e., ζ = 0) or with slightly charged surfaces showed a great tendency to agglomeration, and thus their suspensions were characterized by a low stability. The stronger the charge of the particles, the greater their colloidal stability. When the values of the zeta potential are higher than +30 mV or lower than −30 mV, such a suspension shows a good stability, i.e., the particles do not form agglomerates [40,41].

Here, the arabic gum has been applied as a stabilizing agent which was intended to prevent or at least limit such an agglomeration. In order to determine the stabilizing properties of arabic gum, the zeta potentials of magnetic nanoparticles (obtained previously in Massart synthesis) in aqueous solutions of arabic gum (at concentrations 1.0%, 3.0%, 5.0% and 7.0%, respectively) have been determined. The zeta potential of aqueous suspension of magnetic nanoparticles (without the presence of arabic gum) has also been measured. Results of the performed measurements are shown in Table 1.

The zeta potential of aqueous suspension of magnetic nanoparticles without a stabilizing agent is −15.63 mV. This value may indicate the low stability of such a suspension, resulting in the agglomeration of Fe_3_O_4_ nanoparticles. The results obtained are consistent with the results of the investigations of Schwegmann et al. In their work, the zeta potential for the aqueous suspension of uncoated Fe_3_O_4_ nanoparticles was also approximately −15 mV [42]. In the case of the measurement performed for the suspension of magnetic nanoparticles in an aqueous solution of arabic gum, the zeta potential of such suspension showed more negative values. This may indicate an increase in the stability of the suspension, and thus a direct influence of arabic gum on the process of agglomeration of magnetic nanoparticles. An increase in the stabilizer concentration to 3% resulted in the preparation of a highly stable suspension, as evidenced by its value of the zeta potential less than −30 mV. Further increasing the stabilizer concentration only slightly affected the values of the zeta potential. Therefore, it may be concluded that the effective concentration of the stabilizer, i.e., arabic gum, is 3%, and according to the principles of green chemistry, there is no need to additionally increase the amount of arabic gum due to such small differences in the zeta potential for the next concentrations, i.e., 5% and 7%.

**Figure 4:**
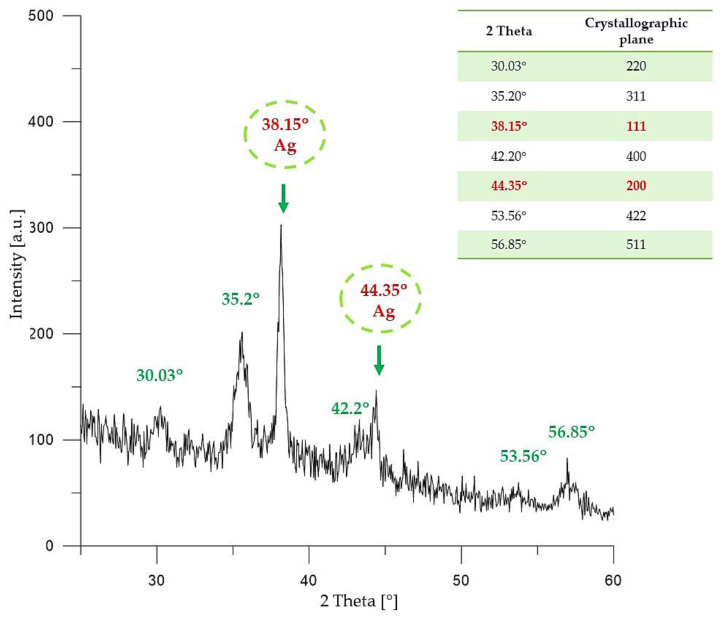
XRD pattern of Fe_3_O_4_@Ag particles.

In Figure 4, results of the investigations on the crystallinity of nanosilver-coated magnetic nanoparticles are presented.

In the XRD pattern shown in Figure 4, the following peaks with 2θ values can be observed: 30.03°; 35.2°; 43.2°; 53.56°; and 56.85°. The mentioned XRD diffraction peaks are characteristic for the crystallographic planes 220, 311, 400, 422 and 511 of iron (II, III) nano-oxide with a cubic structure. Such visible diffraction peaks of iron (II, III) nano-oxide are consistent with data reported in the literature [43,44,45].

Furthermore, on the XRD pattern presented in Figure 4, two diffraction peaks with 2θ values 38.15° (111) and 44.35° (200) can be observed. Such peaks clearly indicate the presence of crystalline metallic silver nanoparticles with a cubic structure (JCPDS Card No. 65–2871).

**Figure 5:**
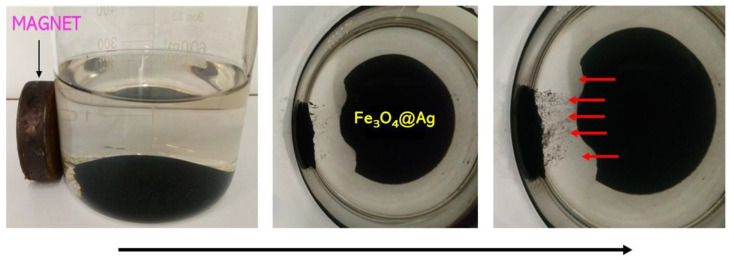
Magnetic properties of the obtained Fe_3_O_4_@Ag particles.

Nanosilver-coated magnetic nanoparticles, as well as uncoated magnetic nanoparticles, exhibited magnetic behavior, i.e., they were able to interact with external magnetic field, which proves that such a coating does not affect these properties. This has been presented in Figure 5.

In the images presented in Figure 5, it is possible to notice that the obtained Fe_3_O_4_@Ag particles are characterized by magnetic properties. The behavior of these particles under the influence of the magnetic field and their spontaneous movement toward this field indicate the mentioned magnetic properties.

**Figure 6:**
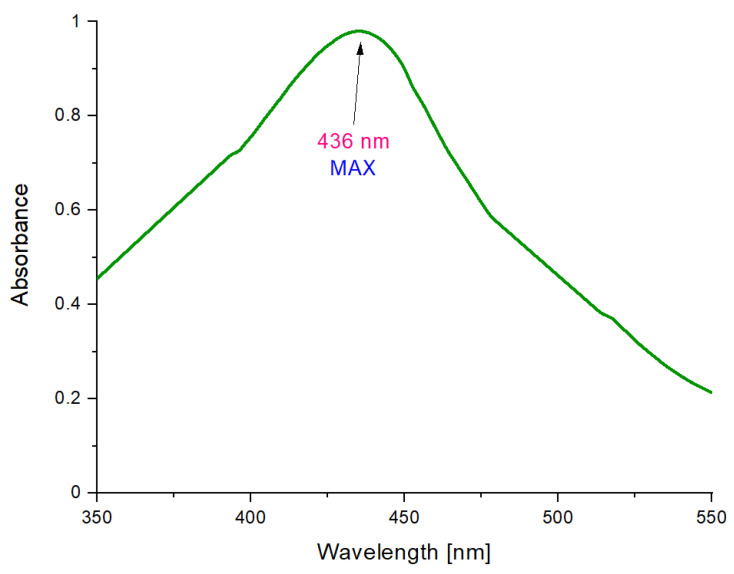
UV–Vis (Ultraviolet Visible Spectroscopy) spectrum of Fe_3_O_4_@Ag particles.

In Figure 6, the UV–Vis spectrum of Fe_3_O_4_@Ag is presented.

The main objective of this investigation was to determine whether the reaction of the reduction of Ag^+^ to Ag^0^ occurred, which would indicate that as a result of the synthesis, nanosilver-coated magnetic nanoparticles have been obtained. The colloidal suspension of silver nanoparticles is characterized by a strong absorption band in the range of ultraviolet and visible radiation, which is caused by the phenomenon of surface plasmon resonance. This collective oscillation and the excitation of the outermost electrons on the surface of nanoparticles resulted in the appearance of a characteristic, intense color of the colloidal solution. The location of the maximum absorption range depends, for example, on the type of nanoparticles, their size, and shape. The maximum absorbance of silver nanoparticles occurs in the range of 380–450 nm [46,47].

On the obtained UV–Vis spectrum, an absorbance maximum at 436 nm can be observed. Therefore, it may be reported that such a value is in the previously mentioned range characteristic for silver nanoparticles. Such an observation clearly indicates the reduction of silver ions into metallic silver (Ag^0^). Silver has deposited on the surface of the magnetic nanoparticles, thus forming their coating. The shift of the absorption bands towards longer wavelengths may be caused by a wide distribution of particles, a larger size of particles, or by their agglomeration. Such a phenomenon may also be due to the charge transfer between nanosilver and iron (II, III) nano-oxide in the resulting coated structure.

**Figure 7:**
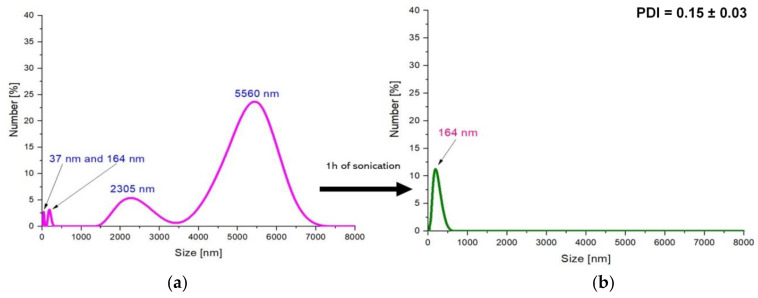
DLS measurements of the Fe_3_O_4_@Ag particles directly after the synthesis (**a**) and after 1 h of sonication (**b**) (PDI- polydispersity index).

**Figure 8:**
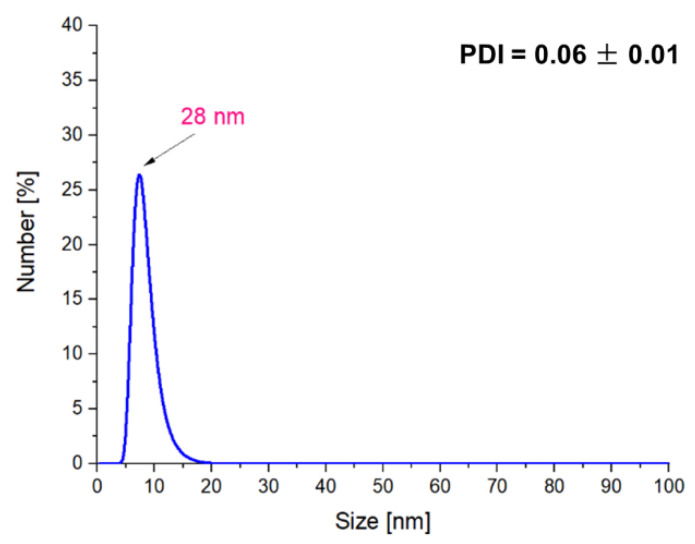
DLS measurements of the Fe_3_O_4_@Ag particles after 2 h of sonication.

In Figure 7 and Figure 8, results of investigations on the sizes of Fe_3_O_4_@Ag particles are presented. The study was performed directly after the synthesis and after the specific periods of treatment of the obtained suspensions with ultrasound energy (i.e., sonication process).

Based on the analysis of the size of particles obtained in the aqueous environment, it was observed that the particles predominantly had a size of 2000–6000 nm in the tested suspension. The investigation of silver-coated Fe_3_O_4_ particles prepared with the use of the stabilizing agent—arabic gum—resulted in the synthesis of materials of micro-, and importantly, nanometric sizes. The predominant sizes of particles in the tested suspension were approximately as follows: 5560 nm; 2305 nm; 164 nm; and 37 nm (Figure 7a). Such results were definitely caused by the application of the stabilizing agent during the synthesis. The operation of the stabilizing agent may, in this case, be defined as the steric stabilization. Arabic gum consists of polysaccharides and glycoproteins. Such compounds may adsorb on the colloidal particles. As a result, structures (e.g., polysaccharides chains) protruding from two adjacent particles prevent them from interactions, thus significantly reducing their agglomeration. Additional effects are based on the electrostatic repulsion of adjacent molecules with the same charge. The accumulation of the electric positive charge on the functional groups (carboxylic or amino ones) occurring in the compounds included in the arabic gum resulted in the repulsion of these groups. Finally, the steric effect caused by the branched compounds from arabic gum adsorbed on the particles keeping them apart from each other was enhanced by additional electrostatic interactions. Therefore, the use of arabic gum as a stabilizing agent reduces magnetic nanoparticle agglomeration to some extent [48,49].

However, because in an aqueous environment nanoparticles tend to strongly agglomerate and form large-sized aggregates having a size of several thousand nanometers, the stabilizing agent applied (i.e., 3% aqueous solution of arabic gum) shows insufficient stabilizing effect. Therefore, in order to disintegrate the agglomerates formed and to obtain smaller particles with a narrower size distribution, a sonication process was performed. After sonication, the obtained particles were also subjected to analysis of their size via the DLS technique.

The treatment of obtained particles by the ultrasound energy for 1 h resulted in the preparation of particles with a size of approx. 163 nm, while the use of this process for the next 1 h resulted in obtaining the nanosized materials (28 nm). Additionally, such a process contributes to the preparation of the monodisperse mixture.

Based on the performed investigations, it was proven that the sonication process, i.e., the disintegration of aggregates of particles (agglomerates) by means of ultrasound energy, is an effective way to break up the particle agglomerates. During the preparation of nanomaterials, the essential parameter is their size influencing their properties; thus, the formation of the particle aggregates is unfavorable from the viewpoint of the potential application of such systems.

**Figure 9:**
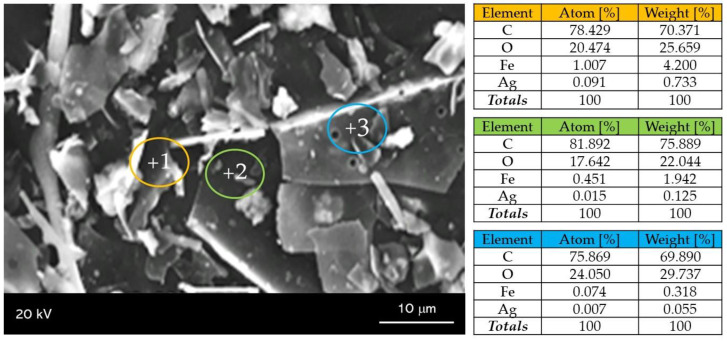
SEM-EDS (Scanning Electron Microscopy with Energy Dispersive Spectroscopy) analysis of Fe_3_O_4_@Ag nanoparticles.

In Figure 9, an SEM (Scanning Electron Microscopy) image of the obtained material and the elemental composition of individual points marked on the microphotograph have been presented.

In Figure 9, the microstructure and the morphology of obtained nanosilver-coated iron (II, III) oxide nanoparticles can be observed. The structure visible on the SEM image does not clearly indicate the preparation of nanomaterials with a spherical shape which was reported in other literature reports [50]. The nanomaterials obtained have a flaked structure, which may be due to the fact that their synthesis was performed in the presence of arabic gum used as the stabilizing agent. EDS (Energy Dispersive Spectroscopy)analysis showed that the composition of the materials received contains only those elements that are part of Fe_3_O_4_@Ag.

**Figure 10:**
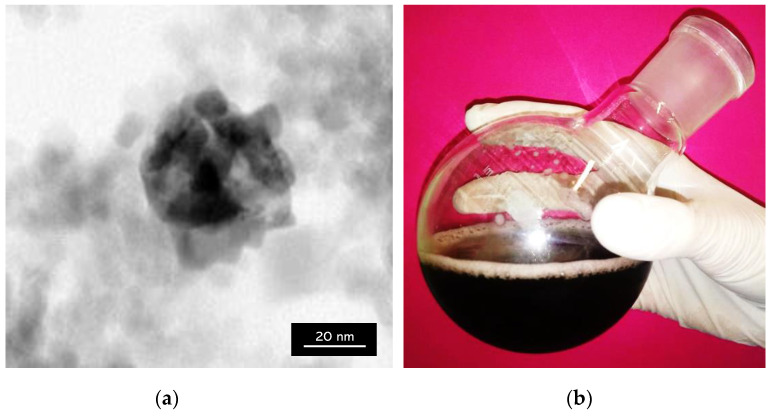
(**a**) TEM (Transmission Electron Microscopy) image of Fe_3_O_4_@Ag nanoparticles; (**b**) Fe_3_O_4_@Ag nanoparticles in suspension.

The analysis verifies whether silver nanoparticles coated the surface of Fe_3_O_4_ nanoparticles. In Figure 10, a TEM image of the obtained materials is presented.

TEM analysis determines the morphology, shape, and size of materials. In the TEM image presented in Figure 10a, the spherical structure of the material core, i.e., Fe_3_O_4_ particles, and the silver nanoparticles visible in a form of a lighter spheres surrounding such a core and forming a coating on the Fe_3_O_4_ particles can be observed. Furthermore, single silver nanoparticles remaining in the suspension which have not been attached into the surface of magnetic nanoparticles have been observed. This is probably caused by too many silver nanoparticles relative to the magnetic ones, resulting in the attachment of only a part of the magnetic core. Moreover, the obtained TEM image confirmed the formation of a core–shell type nanomaterial.

**Figure 11:**
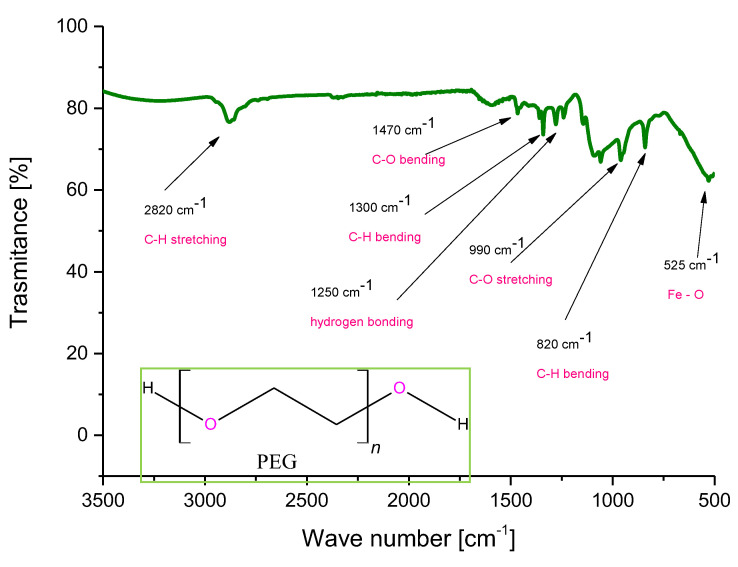
FT-IR spectrum of Fe_3_O_4_@Ag@PEG particles.

Below, in Figure 11, an FT-IR spectrum of Fe_3_O_4_@Ag@PEG particles is shown. The characteristic vibrations observed on the spectra and the corresponding functional groups have been indicated on the spectrum.

In the above figure, the characteristic bands derived from the coating formed on the surface of nanosilver-coated magnetic nanoparticles. The bands corresponding to the chemical bonds characteristic for poly(ethylene glycol) used for forming such a coating have been observed. Moreover, many new bands compared to the FT-IR spectrum of uncoated Fe_3_O_4_ nanoparticles presented in Figure 2 may be observed, which confirms that the reaction relied on the formation of the PEG coating proceeded successfully.

Importantly, bands at approximately 1250 cm^−1^ may indicate the hydrogen bonding as secondary bonding between PEG and the nanoparticles. Such an interaction is formed between the oxygen from the ether group of PEG and the protonated magnetic nanoparticles [51,52]. The formation of the additional coating on the surface of the particles increase their functionality via the possibility of the attachment of active substances to such modified surfaces and, importantly, reduces the agglomeration of these particles which spontaneously tend to form clusters (i.e., they behave like small magnets). The correct course of PEGylation process may be also confirmed, comparing the results of the investigations performed with data in the literature, where the same FT-IR spectra of PEG coated particles have been presented [53]. Thus, the synthesis methodology presented here enables us to conclude that the chosen research direction, as well as the results obtained, are consistent with the scientific trend concerning the formation of core–shell nanoparticles presented by other researchers.

**Figure 12:**
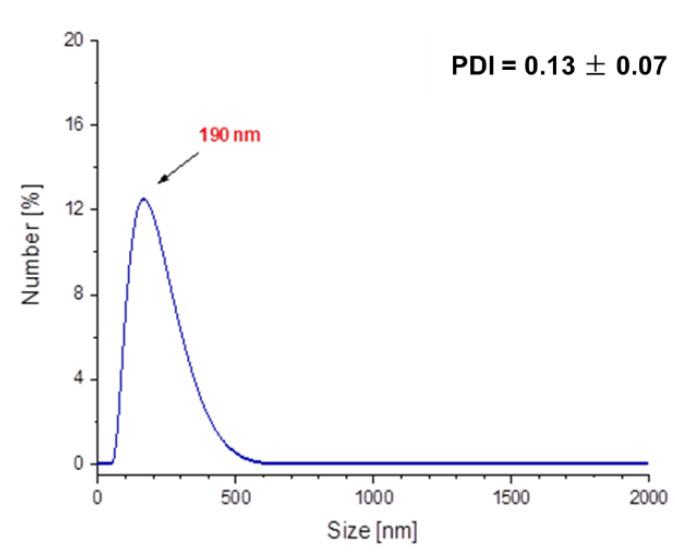
DLS measurement of the Fe_3_O_4_@Ag@PEG particles.

**Figure 13:**
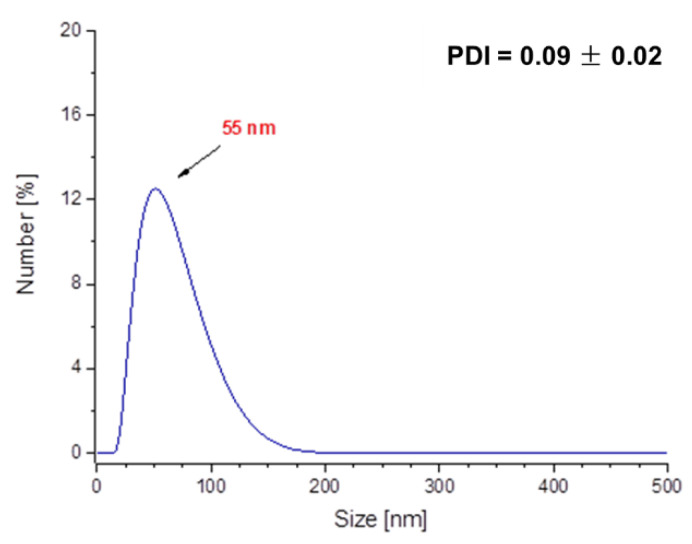
DLS measurement of the Fe_3_O_4_@Ag@PEG particles after 15 min of sonication.

In Figure 12 and Figure 13, results of investigations on the sizes of Fe_3_O_4_@Ag@PEG particles are presented.

The results of DLS analysis of the obtained materials presented in Figure 12 indicates that their size is approximately 190 nm. The obtained particles coated with PEG are characterized by significantly larger sizes compared to those ones without such a shell. Nonetheless, it should be emphasized that PEG-uncoated particles exhibited significantly higher polydispersity (Figure 10a), and as a result they were subjected to the 2 h of the sonication process to disintegrate particles’ agglomerates and obtain the monodisperse suspension. The PEGylated nanosilver-coated particles were characterized by large sizes, although after 15 min of the sonication process, nanoparticles with an average size of approximately 55 nm (Figure 13) were obtained. Therefore, it may be concluded that the PEGylation of particles also enables the obtaining of nanosized materials and, importantly, the time of the sonication process used to disintegrate agglomerates of PEG-coated particles is eight-fold faster compared to the time of this process needed to disintegrate Fe_3_O_4_@Ag without the PEG coating.

## 3. Materials and Methods

### 3.1. Materials

Iron (II) chloride tetrahydrate (98%), iron (III) chloride hexahydrate (97%), hydrochloric acid (36.5–38.0%), poly(ethylene glycol) PEG (Mn = 20,000 g/mol (Mn- Number Average Molecular Weight)) and hydroxylamine hydrochloride (98%) were purchased in Sigma Aldrich (Saint Louis, MO, USA). Sodium hydroxide (pure p.a., 98.8%), silver nitrate (pure p.a., 99.9%) and arabic gum (powder) were bought from Avantor Performance Materials Poland S.A. (Gliwice, Poland).

### 3.2. Preparation of Fe_3_O_4_ Particles Via Massart Synthesis

**Figure 14:**
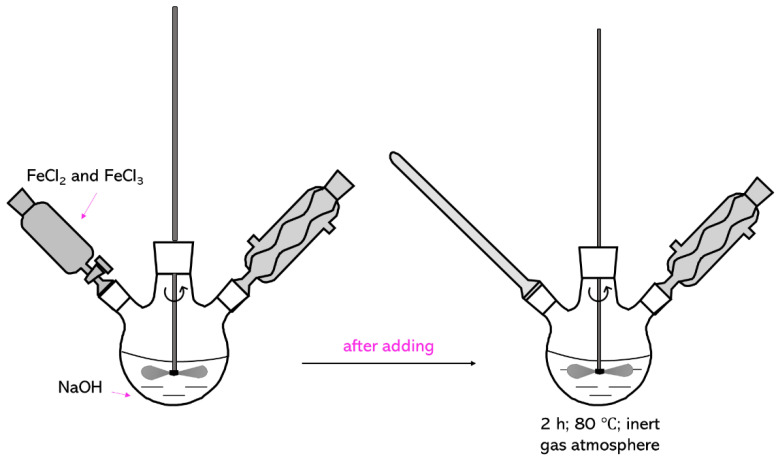
The scheme of Massart synthesis.

Iron oxide particles were obtained via Massart synthesis, i.e., the chemical co-precipitation of iron ions in alkaline environment [54,55]. Firstly, 1.5 M NaOH solution was heated to 80 °C. After reaching this temperature, 25 mL 10 mM solution of FeCl_2_ × 4H_2_O and FeCl_3_ × 6H_2_O in HCl were added dropwise to the reaction mixture and the whole solution was maintained for 2 h at 80 °C with constant stirring and in an inert gas (argon) atmosphere. In Figure 14, the scheme of the Massart synthesis is presented.

**Figure 15:**
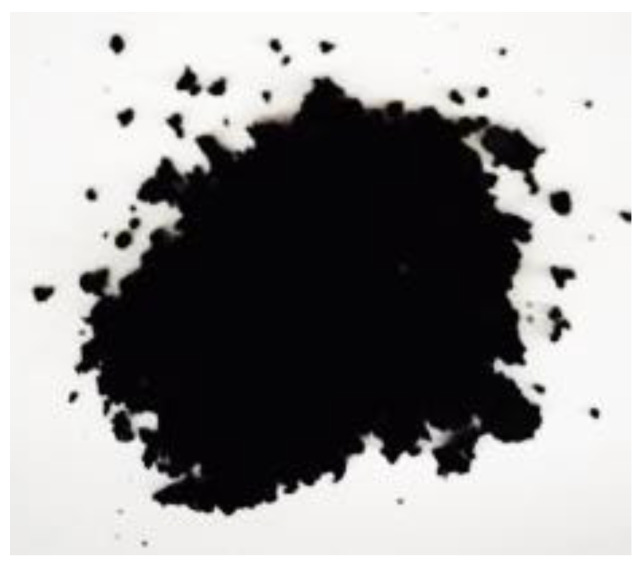
The powdered iron (II, III) oxide nanoparticles.

Next, such obtained Fe_3_O_4_ particles were analyzed. Before the crystallinity and the chemical structure investigations, a certain volume of the magnetic nanoparticle suspension was lyophilized. Black powdered iron (II, III) oxide nanoparticles are shown below in Figure 15.

The iron (II, III) oxide nanoparticles obtained in Massart synthesis were subsequently subjected to the next reaction, aiming for the formation of a nanosilver layer on their surface. Firstly, it was important to determine the stability of the obtained aqueous suspension of magnetic nanoparticles, which enabled verification of the conditions in which further syntheses have been performed.

### 3.3. Investigations on the Stability of the Suspensions of Magnetic Nanopartciles Via the Measurements of the Zeta Potential

Due to the tendency of magnetic nanoparticles to agglomerate, the stability of their various suspensions has been evaluated. The study was performed for different formulations, i.e., for aqueous suspension of nanoparticles as well as their suspensions in aqueous solutions of arabic gum at various concentrations (i.e., 1%, 3%, 5% and 7%). The measurements were conducted at 25 °C using Zetasizer Nano ZS Malvern (Malvern Panalytical Ltd., Malvern, UK). The main purpose of the research was to select the reaction conditions for further syntheses.

### 3.4. Preparation of Nanosilver-Coated Fe_3_O_4_ Particles (Fe_3_O_4_@Ag)

**Figure 16:**
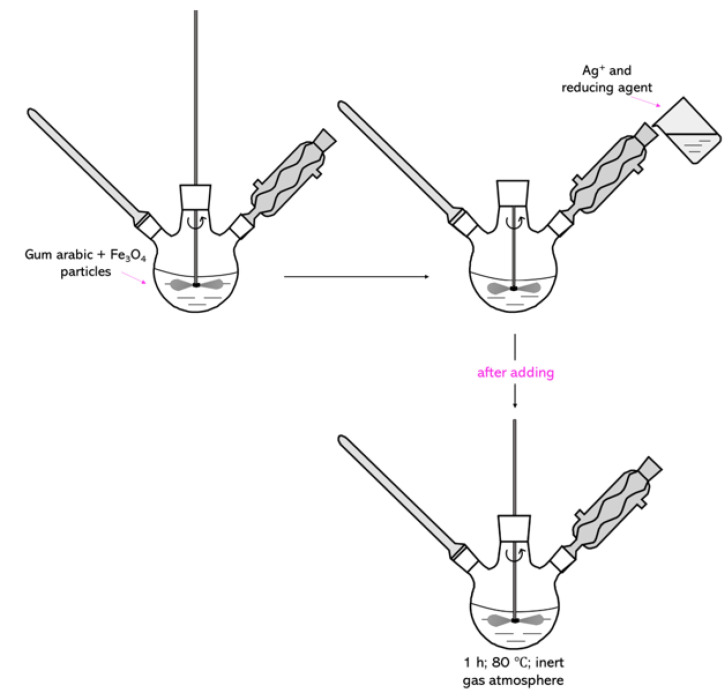
The scheme of Fe_3_O_4_@Ag synthesis.

Firstly, 7.5 mL of Fe_3_O_4_ particle suspension obtained by the Massart synthesis was introduced into a 3% aqueous solution of arabic gum, and the whole mixture was heated to 80 °C. After reaching this temperature, two additional reagents were introduced into the reaction mixture: 0.1 M silver nitrate solution (3.75 mL) which acted as a source of silver ions, and 0.1 M hydroxylamine hydrochloride (1.50 mL) as a reducing agent used to reduce silver ions into metallic silver. After addition of these reagents, the reaction was maintained for 1 h at 80 °C. The whole process was conducted in an inert gas (argon) atmosphere with constant stirring. In Figure 16, the scheme of the synthesis for the formation of the nanosilver coating on Fe_3_O_4_ particles is shown.

Obtained Fe_3_O_4_@Ag particles have been investigated to determine their size, crystallinity, morphology, and the elemental composition.

### 3.5. Analysis of Crystallinity of Particles Obtained

Crystallinity of obtained materials—both Fe_3_O_4_ particles and Fe_3_O_4_@Ag particles—was determined by a wide-angle X-ray diffraction technique. The research was conducted using a Bruker D2 Phaser diffractometer. All measurements were carried out in the reflection mode (kCu = 1.54 Å), at room temperature. The measurement range applied was 5–40° (increment: 0.02.).

### 3.6. FT-IR Analysis of Particles Obtained

Studies were performed to determine the presence of characteristic functional groups in analyzed materials. Analysis was conducted using a Nicolet iS5 Thermo Scientific spectrometer (Thermo Fisher Scientific, Waltham, MA, USA) FT-IR spectra were obtained in the range of 4000–550 cm^−1^.

### 3.7. Analysis of Particles Size by Dynamic Light Scattering (DLS) Method

Particle sizes were measured by dynamic light scattering (DLS) technique using a Zetasizer Nano ZS Malvern. The measurements were performed at 25 °C.

### 3.8. Investigation on the Optical Properties of Fe_3_O4@Ag Particles

In order to verify the presence of nanosilver coating on the surface of Fe_3_O_4_ particles, UV–Vis spectroscopy was performed. The study was conducted by means of a ThermoScientific Evolution 220 UV-Vis spectrometer (Thermo Fisher Scientific, Waltham, MA, USA).

### 3.9. Sonication of the Particles Suspensions

In order to disintegrate the agglomerates of the obtained particles, a sonication process, i.e., the application of sound energy, was employed. For this purpose, an ultrasound Omni Sonic Ruptor homogenizer (parameters: power, 40%; pulsation, 50%) was applied.

### 3.10. Analysis of the Surface Morphology by Scanning Electron Microscopy with Energy Dispersive X-ray Spectroscopy (SEM-EDS Technique)

The surface morphology of obtained particles and their elemental composition were determined using a scanning electron microscope equipped with an EDS detector (voltage 10 kV). The investigation was performed using a Helios NanoLab H50HP FEI microscope (FEI company, Hillsboro, OR, USA). SEM images were obtained at an accelerating voltage of 5 kV.

### 3.11. Analysis of Particles Via Transmission Electron Microscopy

The surface morphology of the obtained materials and their elemental composition were determined using a JEOL JEM1200 (JEOL USA, Inc., Peabody, MA, USA) transmission electron microscope (accelerating tension: 120 kV).

### 3.12. Synthesis and Characterization of PEGylated Nanoparticles

A further step of the synthesis involved the formation of a PEG layer on previously obtained and characterized nanosilver-coated magnetic nanoparticles. For this purpose, 8 mL of nanosilver-coated magnetic nanoparticles in suspension after the sonication process were introduced into 100 mL of 1% PEG solution. The reaction was performed at 60 °C at constant stirring and in an inert gas atmosphere. The reaction time was 1 h.

Obtained structures have been subjected to FT-IR spectroscopy to characterize their chemical structure, and via the DLS technique to verify their size. The equipment applied for the investigations as well as the measurements’ conditions was the same as previously.

## 4. Conclusions

Massart synthesis leads to the preparation of iron (II, III) oxide with a cubic structure that was proved by diffraction peaks visible on the obtained XRD pattern. Additionally, on the FT-IR spectrum, bands at 539 cm^−1^ and 420 cm^−1^ corresponding to the stretching vibrations of Fe–O were observed, which also confirmed the synthesis of Fe_3_O_4_ particles.

In the research, nanosilver-coated magnetic nanoparticles have been obtained. This was confirmed by diffraction peaks with 2θ values 38.15° and 44.35°, which clearly indicate the presence of crystalline metallic silver nanoparticles with a cubic structure on the surface of magnetic nanoparticles. Additionally, suspension of coated particles exhibited an absorbance maximum at 436 nm which is characteristic for silver nanoparticles.

Iron (II, III) oxide nanoparticles exhibit magnetic properties that, on the one hand cause their spontaneous movement toward an applied external magnetic field, and on the other hand result in their tendency to form agglomerates.

The sound energy-using sonication process contributes to the disintegration of the particle aggregates. The suspension obtained via the Massart process contained particles with a size within the range of 2000–6000 nm. After 15 min sonication, the particle sizes were 800–7000 nm.

Arabic gum used as a stabilizing agent limits the agglomeration of iron (II, III) oxide nanoparticles and leads to the synthesis of particles with a narrower size distribution. This is due to the steric effect caused by the branched compounds from arabic gum adsorbed on the particles keeping them too far from each other to interact. Such an effect is additionally enhanced by the accumulation of an electric positive charge on the functional groups (carboxylic or amino) occurring in the compounds included in the arabic gum, which results in the repulsion of these groups.

The process of PEGylation of the nanosilver-coated magnetic nanoparticles allows the obtaining of suspensions of particles with lower polydispersity, and thus shortens the time of the sonication process, which is necessary to achieve nanometric dimensions of the obtained materials.

The PEGylation of the particles significantly limits their agglomeration, and, importantly, increases their functionality via the possibility of the attachment of various active substances to PEG-coated nanoparticles. Such structures containing the active substance due to the magnetic core may be used in targeted drug delivery systems where the drugs are delivered to the desired place using an external magnetic field.

## Figures and Tables

**Table 1:** Zeta potential measurements for various suspensions of magnetic nanoparticles.

Type of Tested Magnetic Nanoparticles Suspension	Zeta Potential (mV)
Aqueous suspension, without stabilizing agent	−15.63
1% arabic gum solution	−24.98
3% arabic gum solution	−31.05
5% arabic gum solution	−31.78
7% arabic gum solution	−31.32

## Sample Availability

Samples of the compounds are available from the corresponding authors.
